# Global research trends in transcranial magnetic stimulation for stroke (1994–2023): promising, yet requiring further practice

**DOI:** 10.3389/fneur.2024.1424545

**Published:** 2024-08-29

**Authors:** Xin-Yu Li, Rong Hu, Tian-Xiao Lou, Yang Liu, Ling Ding

**Affiliations:** Department of Rehabilitation and Traditional Chinese Medicine, Institute of Rehabilitation and Health Care, Hunan College of Traditional Chinese Medicine, Zhu Zhou, China

**Keywords:** bibliometric analysis, transcranial magnetic stimulation, stroke, dysphagia, cognitive impairment, research trends, hotspots

## Abstract

**Background:**

Scholars have been committed to investigating stroke rehabilitation strategies over many years. Since its invention, transcranial magnetic stimulation (TMS) has been increasingly employed in contemporary stroke rehabilitation research. Evidence has shown the significant potential of TMS in stroke research and treatment.

**Objective:**

This article reviews the research conducted on the use of TMS in stroke from 1994 to 2023. This study applied bibliometric analysis to delineate the current research landscape and to anticipate future research hotspots.

**Method:**

The study utilized the Web of Science Core Collection to retrieve and acquire literature data. Various software tools, including VOSviewer (version 1.6.19), CiteSpace (version 6.3.R1), Scimago Graphica (version 1.0.36), and WPS (version 11572), were used for data analysis and visualization. The review included analyses of countries, institutions, authors, journals, articles, and keywords.

**Results:**

A total of 3,425 articles were collected. The top three countries in terms of publication output were the United States (953 articles), China (546 articles), and Germany (424 articles). The United States also had the highest citation counts (56,764 citations), followed by Germany (35,211 citations) and the United Kingdom (32,383 citations). The top three institutions based on the number of publications were Harvard University with 138 articles, the University of Auckland with 81 articles, and University College London with 80 articles. The most prolific authors were Abo, Masahiro with 54 articles, Fregni, Felipe with 53 articles, and Pascual-Leone, Alvaro with 50 articles. The top three journals in terms of article count were *Neurorehabilitation and Neural Repair* with 139 articles, Clinical *Neurophysiology* with 128 articles, and *Frontiers in Neurology* with 110 articles. The most frequently occurring keywords were stroke (1,275 occurrences), transcranial magnetic stimulation (1,119 occurrences), and rehabilitation (420 occurrences).

**Conclusion:**

The application of TMS in stroke research is rapidly gaining momentum, with the USA leading in publications. Prominent institutions, such as Harvard University and University College London, show potential for collaborative research. The key areas of focus include post-stroke cognitive impairment, aphasia, and dysphagia, which are expected to remain significant hotspots in future research. Future research should involve large-scale, randomized, and controlled trials in these fields. Additionally, identifying more effective combined therapies with rTMS should be a priority.

## 1 Introduction

Stroke, a devastating medical condition, has a profound impact on human wellbeing. According to Gorelick, stroke had the highest prevalence in Asia, followed by Eastern Europe and Central Latin America (1). In the United States, ~800,000 individuals are affected by stroke annually (2). The average lifetime cost for each stroke patient was reported to be $140,048 as of 2014 (3). In China, there were 3.94 million new stroke cases, 28.76 million prevalent cases, and 2.19 million deaths attributed to stroke in 2019 (4). An analysis of economic burden revealed that ~3–4% of healthcare expenditure in Western nations is allocated to stroke care (5). Moreover, stroke is a leading cause of long-term disability in the United States, affecting 26% of newly diagnosed patients (6).

Consequently, significant efforts have been directed toward developing rehabilitation and research strategies aimed at addressing the multifaceted challenges of disability, dysfunction, and the economic burden associated with stroke. The diverse range of dysfunction resulting from stroke necessitates a variety of rehabilitation and treatment approaches, with transcranial magnetic stimulation (TMS) emerging as one of the promising methods in this field.

In 1985, Barker et al. pioneered the use of TMS on cortical areas, successfully inducing movement in the contralateral hand or foot, marking the advent of TMS technology (7). Initially, TMS technology was employed as a novel tool for investigating brain function, and it was primarily used to probe the physiological mechanisms underlying stroke and other neurological conditions (8). Subsequently, repetitive transcranial magnetic stimulation (rTMS), a specific mode of TMS, gained prominence in stroke rehabilitation research due to its ability to modify and regulate cortical activity beyond the stimulation period, showing potential as a treatment for neurological disorders. In current times, rTMS is recognized as one of the key rehabilitation modalities for stroke (9). Furthermore, recent years have witnessed an increase in studies focusing on rTMS in the context of stroke rehabilitation (10, 11).

Understanding the key areas of focus and developmental trends in TMS for stroke rehabilitation poses a challenge for new researchers. Therefore, it is essential to actively identify emerging research trends and key areas of interest in this field.

Bibliometrics aims to assist new researchers in comprehending research trends and current hotspots through quantitative and qualitative analysis of literature data. By employing data visualization techniques, bibliometrics allows for a comprehensive analysis of the literature within a database, facilitating comparisons of research focus and collaboration across various countries, institutions, and authors (12). Thus, this review aims to enhance the understanding of evolving trends and significant research hotspots in TMS for stroke, particularly for new researchers.

## 2 Materials and methods

### 2.1 Eligibility criteria

This systematic review was conducted in accordance with the PRIBA guidelines (13), ensuring methodological rigor and transparency.

Population: Individuals globally affected by stroke.

Intervention: Transcranial magnetic stimulation (TMS).

Comparators: Various aspects, including the analysis of articles, institutions, citations, author contributions, journals, and keywords.

Outcomes: Detailed outcomes are presented in the subsequent sections on Results and Discussion.

Study design: A range of trials and review articles were included, with a focus on those categorized as “article” or “review article.”

### 2.2 Database

We selected the Clarivate Analytics Web of Science Core Collection (WoSCC), version 2024, as the primary source for our database queries. This database offers extensive citation coverage across a diverse array of core journals, encompassing global research fields such as natural sciences, engineering technology, and biomedicine. It provides comprehensive literature coverage.

### 2.3 Searching strategy

Initially, we accessed the Web of Science (WoS) platform and navigated to the “WoS Core Collection” and the “Science Citation Index Expanded,” covering the period from 1994 to the present. Subsequently, we selected “article” and “review article” as the document types after entering the search query using the following searching subject terms: topic = (“stroke” OR “cerebrovascular accident” OR “hemiparesis” AND “transcranial magnetic stimulation” OR “TMS” OR “rTMS”) (14, 15). We excluded the literature published in 2024 and 2025, including non-English literature. Finally, we downloaded the citation report and literature data on the same day, with the research deadline set to 16 March 2024, to ensure consistency and prevent any changes due to data updates.

### 2.4 Data analysis

First, we selected two researchers to download the data following the same search query mentioned above. The data were not confirmed until two researchers had agreed on the same number of articles to include and to exclude. Second, we used WPS software (version 11572) to analyze the statistical data of top-cited or productive authors, countries, publications, journals, and institutions. Third, we conducted a unified merger for different names of the same country or institution to ensure the accuracy and repeatability of the data. For example, America and the United States of America were merged as the USA. We merged different expressions of the same term for uniformity, such as transcranial magnetic stimulation was merged as TMS. The criteria were saved as a txt document named “same meaning words” and applied in VOSviewer software.

### 2.5 Data visualization

VOSviewer, a prevalent tool in bibliometric analysis, was employed to visualize the data. It encompassed co-authorship analysis, co-occurrence analysis, citation, and co-citation analysis. Co-authorship analysis examined the number of jointly completed articles to analyze relationships between authors, countries, or institutions. Co-occurrence analysis quantitatively assessed relationships between different projects based on their occurrence together. Co-citation analysis assessed the degree of interconnection between cited works by quantifying the frequency with which they are cited together.

We used VOSviewer software (version 6.19) to visually analyze the literature. The software applied preset values as thresholds during the analysis. Subsequently, the software independently selected and retained the analysis results after removing information with a very low degree of association. We employed CiteSpace (version 6.3.R1) to analyze institutions, authors, co-cited authors, and VOSviewer. We also employed the software Scimago Graphica (version 1.0.36). It was used to examine the collaboration among countries and visualize the global distribution of the world map.

### 2.6 Research ethics

Ethical approval was not sought as the data for this review was obtained from a publicly accessible database, aligning with ethical guidelines for secondary data analysis.

### 2.7 The utilization of AI

We have employed Kimi AI (Version: moonshot-v1-20240416), which is a large language model. The application of this process was limited to the entire body of the article, excluding the reference section, for checking mistakes and refining the intended meaning accurately. The refinement procedure was conducted in a collaborative manner, alternating between the AI and the authors, to ensure the accuracy and integrity of the content.

## 3 Result

### 3.1 Global trend of publications and citations

**Figure 1:**
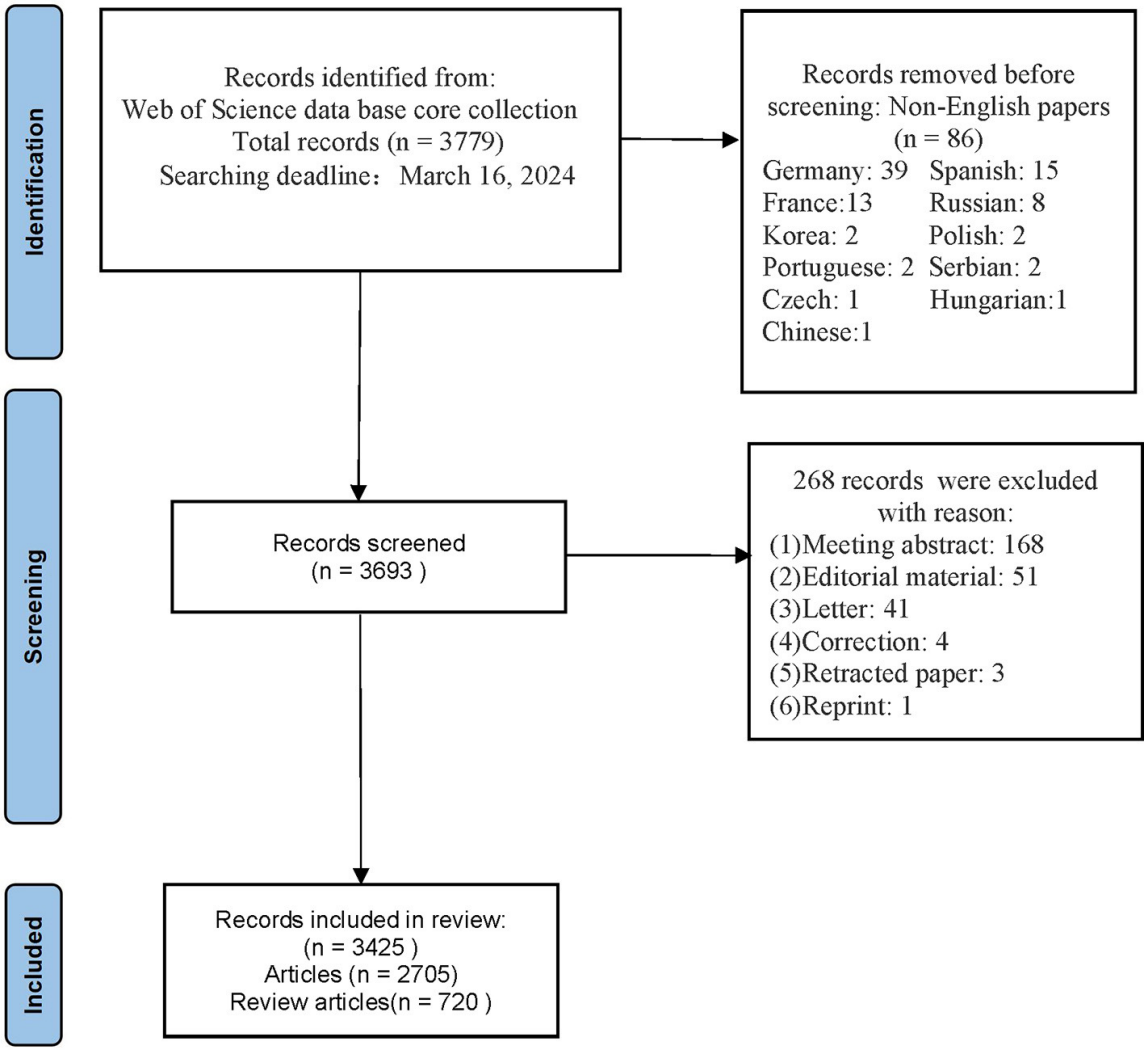
Flow chart of literature inclusion and exclusion.

**Figure 2:**
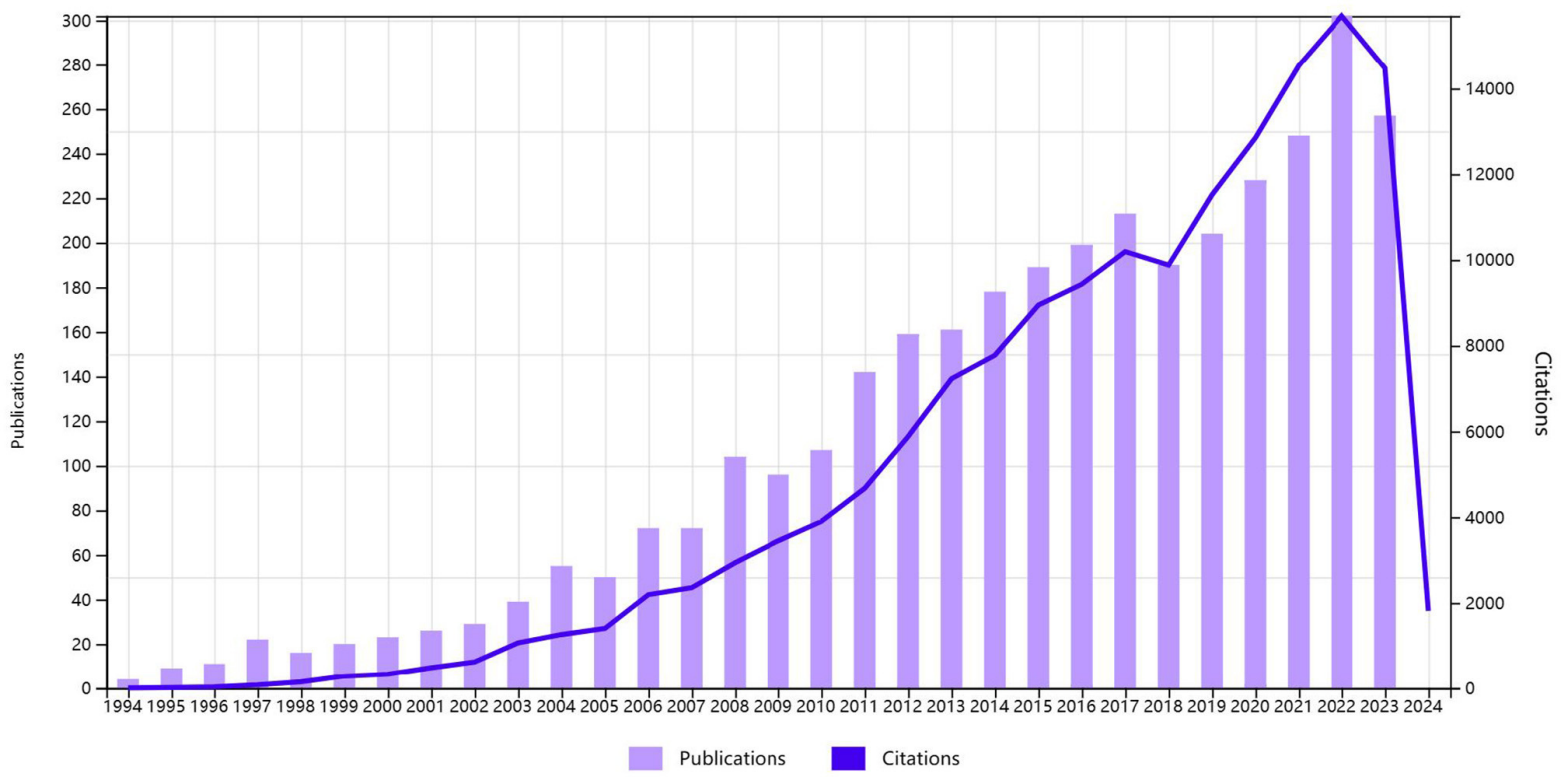
Global trend of publications and total citations on TMS research in stroke from 1994 to 2023.

By 16 March 2024, our search yielded a comprehensive collection of 3,425 relevant articles. Figure 1 illustrates the study inclusion and exclusion criteria. Figure 2 presents the temporal distribution of publications from 1994 to 2023. Notably, the number of publications related to TMS in stroke generally showed an upward trajectory despite small declines in publication numbers during the years 2008–2009, 2017–2018, and 2022–2023. As of the retrieval date, these articles had accumulated a total of 155,407 citations, averaging 45.37 citations per paper, with an H-index of 171, indicating a significant impact in the field.

### 3.2 Analysis of top productive countries

**Figure 3:**
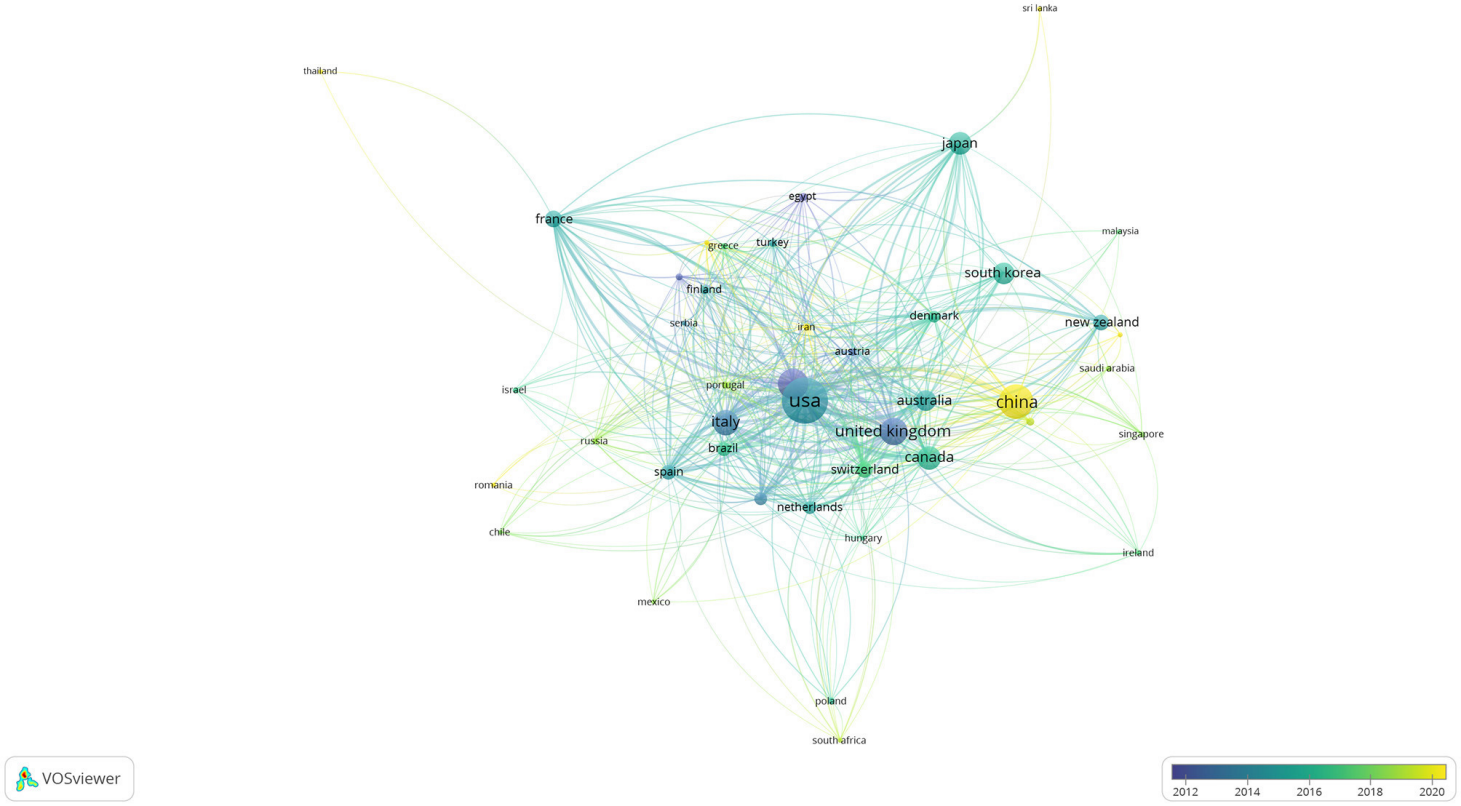
Overlay visualization of countries' literature burst.

**Figure 4:**
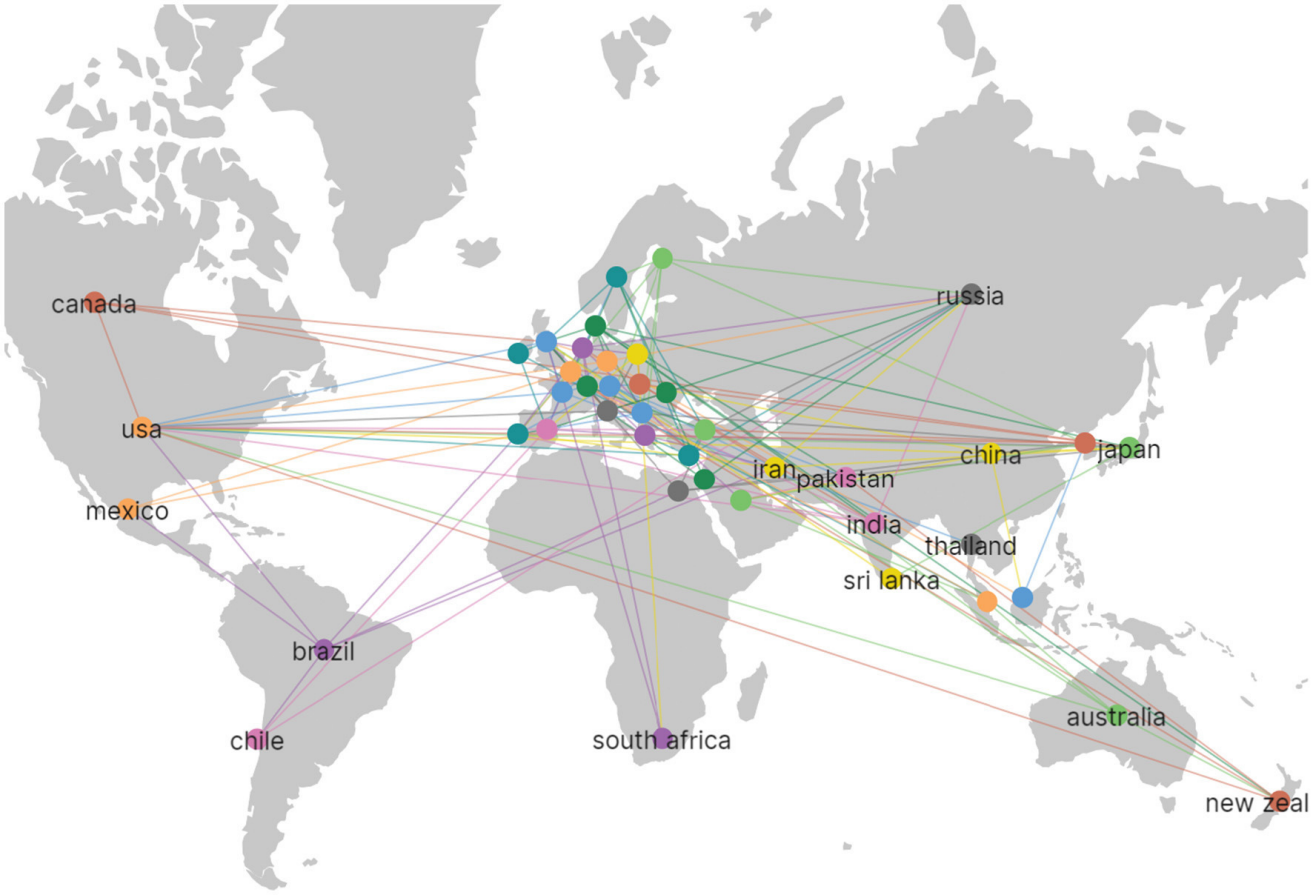
It is the distribution map of the countries. The line between countries represents the cooperation among countries.

Publications in the field of TMS in stroke were contributed by a total of 75 countries. A total of 43 countries met the threshold, as shown in Figure 3, when the threshold was set at five articles for each country. The United Kingdom and Germany were pioneers in TMS research for stroke, with a notable surge in publications in 2012. Italy and the USA experienced a burst of literature around 2014–2016. China experienced a burst of literature around 2020. The USA ranked first with 56,764 citations, followed by Germany with 35,211 citations, and the UK with 32,383 citations, as shown in Table 1. The USA ranked first with 953 articles in terms of publication, followed by China with 546 articles, and Germany with 424 articles, which are among the top three productive countries, as shown in Table 1. Studies of TMS in the field of stroke were more focused on Europe. In the Americas, it was mainly distributed in the USA, Canada, Mexico, Brazil, Chile, and other countries. In Asia, it was mainly distributed in Russia, China, South Korea, and Japan. Oceania was mainly represented by Australia and New Zealand, and Africa was mainly represented by the Republic of South Africa, as shown in Figure 4.

**Table 1:** The top 10 productive countries/regions related to TMS in stroke.

**Rank**	**Country**	**Counts**	**Total citations**	**Average citation/article**	**Total link strength**
1	USA	953	56,764	59.56	695
2	China	546	7,767	14.23	139
3	Germany	424	35,211	83.04	450
4	United Kingdom	343	32,383	94.41	405
5	Italy	306	22,344	73.02	370
6	Canada	254	11,068	43.57	274
7	Japan	242	9,906	40.93	145
8	South Korea	211	4,627	21.93	61
9	Australia	203	11,123	54.79	283
10	France	131	8,863	67.66	215

For Figure 3, the size of the circle reflects the number of publications. The connection between the circles reflects the number of citations. The thicker the connection, the more citations it receives, and the color of the circles focuses on different countries according to the scale in the right corner.

### 3.3 Contributions of top institutions

Among the 3,127 institutions, 424 institutions met the threshold of at least five publications. Table 2 demonstrates that the USA held four out of the top 10 positions. The top three institutions for the number of publications were Harvard University (138 articles), the University of Auckland (81 articles), and University College London (80 articles). The top three institutions in citation ranking were Harvard University (12,813), the National Institute of Neurological Disorders and Stroke (NINDS) (9,621), and University College London (7,506).

**Table 2:** The top 10 institutes in the publications concerning the research of TMS in stroke.

**Rank**	**Institution**	**Country**	**Counts**	**Total citations**	**Total link strength (TLS)**	**Centrality**
1	Harvard University	USA	138	12,813	321	0.13
2	University Auckland	New Zealand	81	4,860	103	0.05
3	University College London	UK	80	7,506	134	0.18
4	National Institute of Neurological Disorders and Stroke (NINDS)	USA	65	9,621	155	0.09
5	Yeungnam University	Korea	62	1,201	17	0.00
6	Emory University	USA	61	2,305	122	0.07
7	University Tubingen	Germany	60	6,910	95	0.07
8	JiKei University	Japan	59	1,176	46	0.02
9	Northwestern University	USA	58	2,131	113	0.03
10	University Manchester	UK	58	4,159	57	0.01

**Figure 5:**
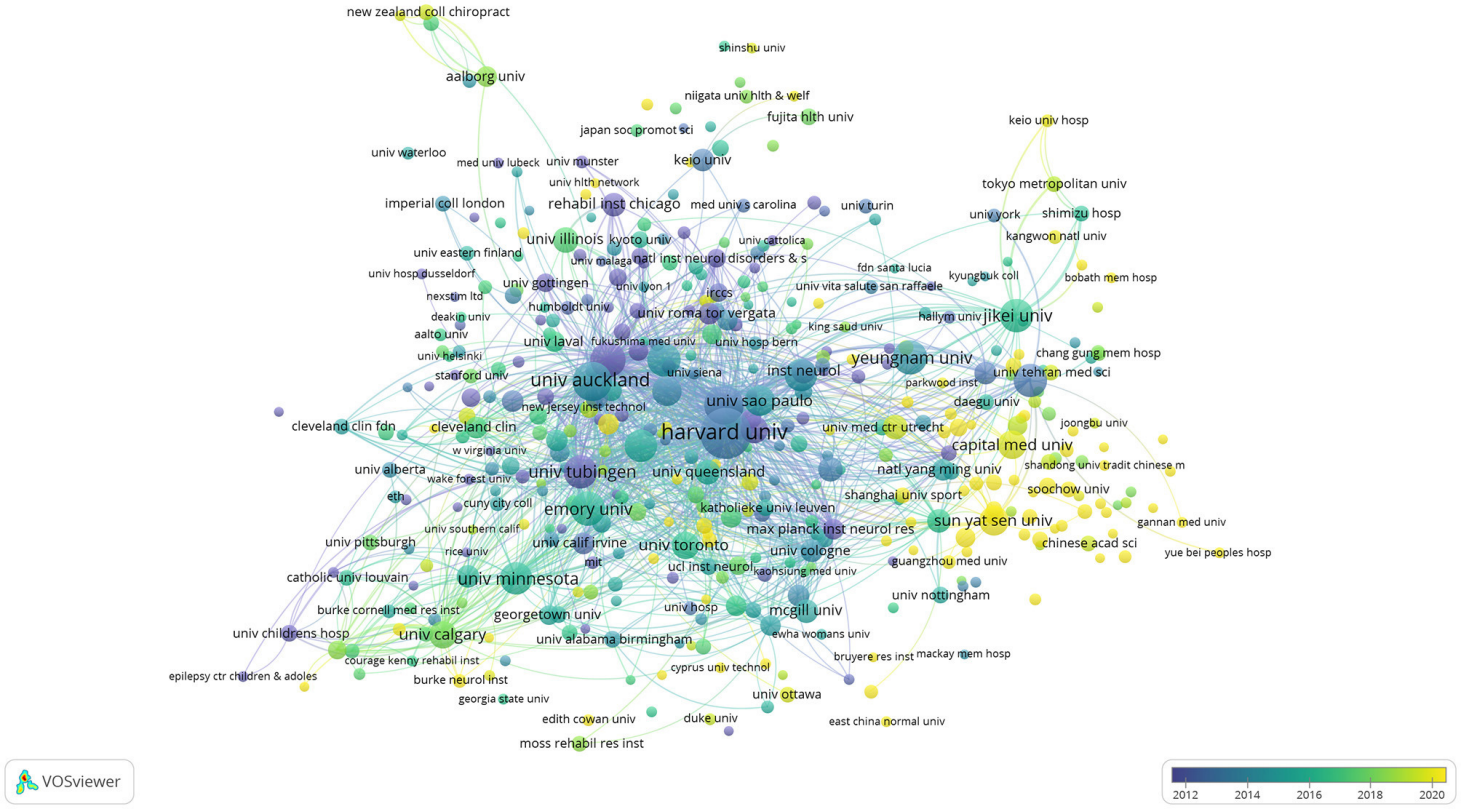
The annual distribution of the publishing institutions is visualized using VOSviewer.

**Figure 6:**
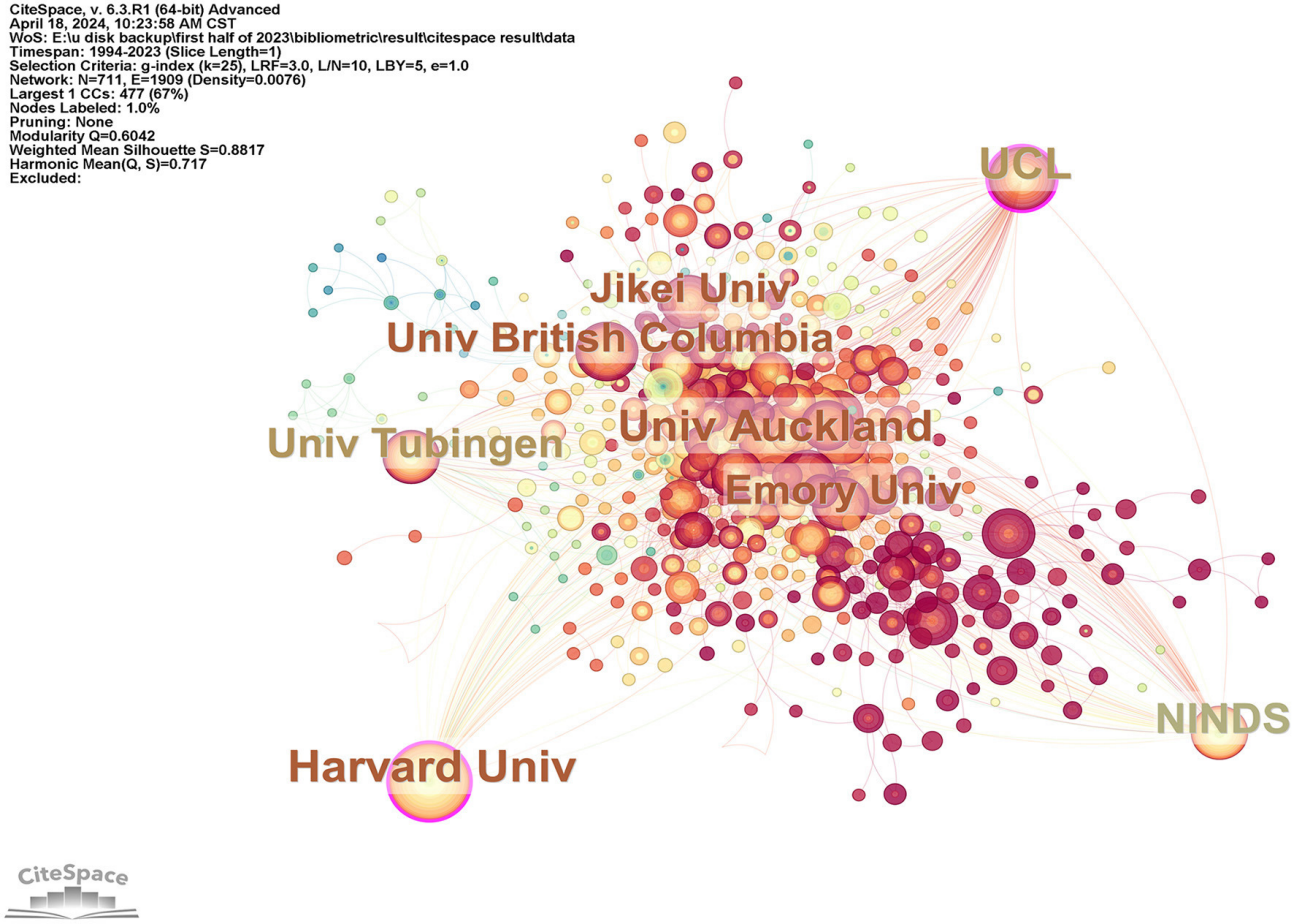
It is an institution visualization generated by CiteSpace, and purple circles in the visualization indicate that the centrality of institutions is >0.1.

Figure 5 shows that 424 institutions have published more than five articles. It also indicated the years during which there was a significant increase in publications from these institutions. The USA experienced a notable surge in literature in 2014, while the National Institute of Neurological Disorders and Stroke (NINDS) saw a similar burst in 2010. Both Harvard University and University College London demonstrated significant betweenness centralities (equal to or >0.1) in the centrality analysis presented in Figure 6. University College London exhibited the highest betweenness centrality at 0.18, highlighting its pivotal role in the research network.

### 3.4 Analysis of authors and co-cited authors

**Figure 7:**
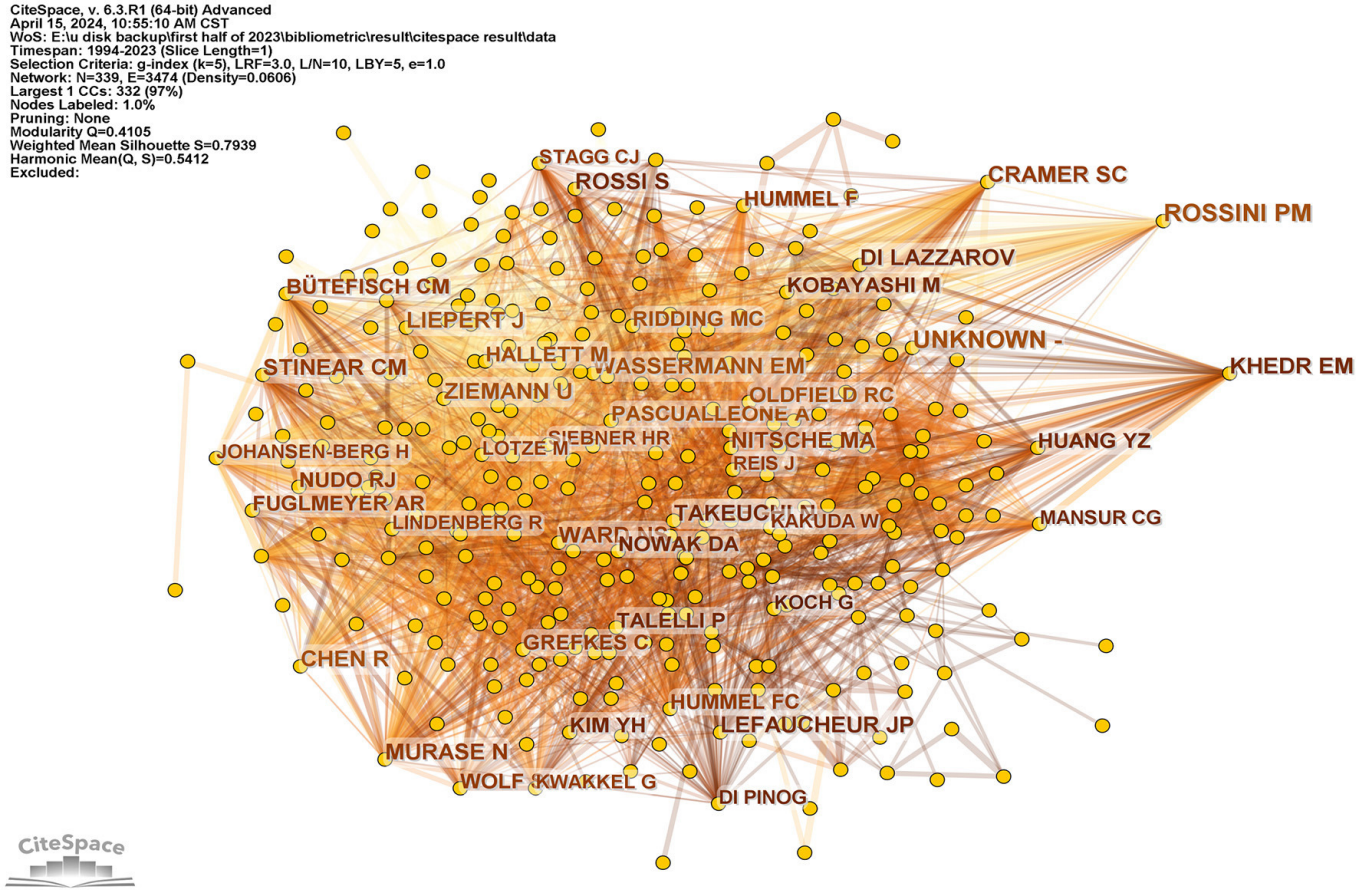
It is a co-cited author visualization picture generated based on CiteSpace. Three co-cited authors whose centrality is over 0.1 are at the right of the picture, including Cramer, SC, Rossni, PM, and Khedr, EM.

The analysis comprised a total of 12,306 authors. A total of 491 authors surpassed the threshold of having published at least five articles, highlighting their substantial contributions. The top three authors with the most publications were Abo, Masahiro (54 articles), Fregni, Felipe (53 articles), and Pascual-Leone, Alvaro (50 articles), as indicated in Table 3. The top three authors in terms of citation count were Pascual-Leone, Alvaro (5,361 citations), Fregni, Felipe (4,973 citations), and Cohen, Leonardo G. (4,425 citations). Nitsche, Ma was co-cited 1,258 times. Liepert, J was co-cited 1,123 times, and Rossini, PM was co-cited 1,049 times among the co-cited authors. Rossini, PM, and Khedr, EM had centrality values of 0.10 when the G-index was set at 5, as shown in Figure 7. It indicated their strong recognition of literature in terms of co-citation. The S value was 0.7939, and the Q value was 0.4105, suggesting a good clustering effect and network homogeneity. This showed that the network was well-connected. However, the density value was 0.0606. No individual reached the betweenness centrality in terms of author centrality. The low betweenness centrality values indicated a need to strengthen cooperation between authors.

**Table 3:** The 10 most productive authors and the top 10 co-cited authors with the highest citations.

**Rank**	**Author**	**Country**	**Counts**	**Citations**	**Co-cited author**	**Country**	**Co-citations**	**Centrality**
1	Abo, Masahiro	Japan	54	1,080	Nitsche, Ma	Germany	1,258	0.07
2	Fregni, Felipe	USA	53	4,973	Liepert, J	Germany	1,123	0.06
3	Pascual-Leone, Alvaro	USA	50	5,361	Rossini, PM	Italy	1,049	0.10
4	Jang, Sung Ho	South Korea	48	589	Ziemann, U	Germany	943	0.04
5	Byblow, Winston D.	New Zealand	46	3,280	Di Lazzaro, V	Italy	915	0.04
6	Cohen, Leonardo G.	USA	46	4,425	Ward, NS	UK	868	0.06
7	Stinear, Cathy M.	New Zealand	42	3,470	Khedr, EM	Egypt	866	0.10
8	Kirton, Adam	Canada	34	1,035	Fregni, Felipe	USA	799	0.09
9	Rothwell, John C.	UK	31	3,689	Stinear, CM	New Zealand	794	0.04
10	Kakuda, Wataru	Japan	31	894	Lefaucheur, JP	France	746	0.06

### 3.5 Contributions of top journals

Table 4 highlights the top three journals by publication volume: *Neurorehabilitation and Neural Repair* (139 articles), *Clinical Neurophysiology* (128 articles), and *Frontiers in Neurology* (110 articles). The top three journals with the most citations were *Clinical Neurophysiology* (10,544 citations), *Brain* (10,128 citations), and *Stroke* (9,724 citations).

**Table 4:** The top 10 journals related to the research of TMS in stroke.

**Rank**	**Journal title**	**Country**	**Counts**	**IF**	**JCR**	**H-index**	**Total citation**
1	Neurorehabilitation and Neural Repair	USA	139	4.2	Q1	121	7,529
2	Clinical Neurophysiology	UK	128	4.7	Q2	200	10,544
3	Frontiers in Neurology	Switzerland	110	3.4	Q2	91	1,326
4	Frontiers in Human Neuroscience	Switzerland	99	2.9	Q3	144	2,886
5	Restorative Neurology and Neuroscience	Netherlands	97	2.8	Q3	83	3,597
6	Stroke	USA	69	8.4	Q1	343	9,724
7	Brain Stimulation	USA	66	7.7	Q1	99	3,803
8	Frontiers in Neuroscience	Switzerland	64	4.3	Q2	144	560
9	Experimental Brain Research	Germany	60	2	Q4	182	3,207
10	Brain	UK	50	14.5	Q1	365	10,128

### 3.6 Analysis of top cited references and co-citation references

In Supplementary Tables 1, 2, a total of 1,618 references met the threshold of 20 citations. The top three articles in terms of citations were those of Langhorne, P. et al. (1,578 citations), Winstein, C. J. et al. (1,555 citations), and Lefaucheur, J. P. et al. (1,289 citations). The total number of co-citations reached 80,636. A total number of 1,361 references reached the threshold of 20 co-citations. The top 3 references in terms of co-citation were Murase, N. et al. (499 co-citations), Rossi, S. et al. (448 co-citations), and Rossini, P. M. et al. (371 co-citations). The top three in terms of total link strength were Murase, N. et al. (15,764), Takeuchi, N. et al. (9,710), and Hummel, F. et al. (9,279).

### 3.7 Co-occurrence analysis of keywords

**Figure 8:**
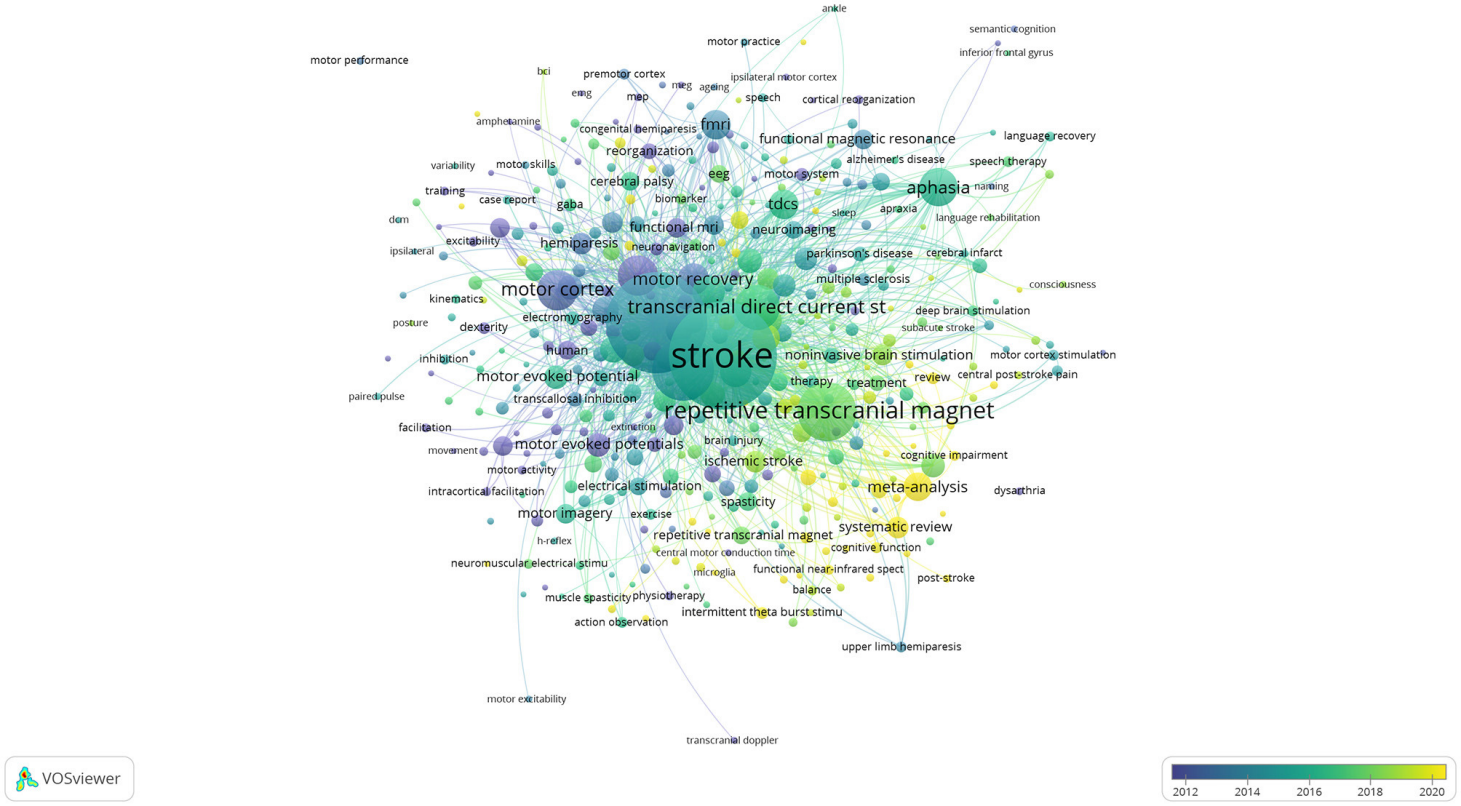
Overlay visualization map of keywords generated based on VOSviewer.

The co-occurrence analysis included 4,355 “author keywords” with 397 keywords meeting the threshold of at least five occurrences. Table 5 presents the top three keywords with the highest frequency: “stroke” (1,275 times), “transcranial magnetic stimulation” (1,119 times), and “rehabilitation” (420 times). Figure 8 illustrates the overlay visualization of keywords. It showed that studies on “cortical stimulation,” “intracortical inhibition,” and “magnetic stimulation” were focused around the year 2012. Research focus shifted to “transcranial magnetic stimulation” and “functional magnetic resonance” from 2012 to 2016. The focus turned to “stroke” and “aphasia” during the period around 2016 subsequently. The research focused on “repetitive transcranial magnetic stimulation,” “dysphagia,” and “non-invasive brain stimulation” around 2018. The latest research focused on “high-frequency repetitive trans” and “cognitive function” around 2020.

**Table 5:** The top 20 keywords with the highest frequency related to the research of TMS in stroke.

**Rank**	**Keyword**	**Occurrence**	**TLS**	**Rank**	**Keyword**	**Occurrence**	**TLS**
1	Stroke	1,275	4,070	11	Neurorehabilitation	109	398
2	Transcranial magnetic stimulation	1,119	3,392	12	fMRI	96	350
3	Rehabilitation	420	1,494	13	tdcs	95	353
4	Repetitive transcranial magnetic stimulation	384	1,128	14	Meta-analysis	93	310
5	Transcranial direct current stimulation	225	773	15	Non-invasive brain stimulation	87	300
6	Motor cortex	182	602	16	Neuromodulation	84	300
7	Plasticity	179	631	17	Cortical excitability	80	291
8	Aphasia	160	551	18	Recovery	80	166
9	Neuroplasticity	117	436	19	Stroke rehabilitation	78	235
10	Motor recovery	113	393	20	Motor function	69	259

## 4 Discussion

The discussion was organized into two main sections and was structured as follows. The first section involved a review of TMS applications in stroke (4.1), where we explored the use of TMS for addressing various post-stroke dysfunctions, such as motor impairments, aphasia, dysphagia, and cognitive impairment. The second part presented the bibliometric analysis of TMS in stroke research (4.2), where various aspects, such as the involvement of countries, institutions, authors, journals, and keywords were examined. Analysis revealed several research hotspots. Based on a thorough analysis of both sections, the conclusion was formulated.

### 4.1 A review of rTMS in the application of stroke

TMS is a good option for non-invasive brain stimulation, but when it comes to stroke rehabilitation, rTMS, which is a more advanced mode of TMS developed 4–8 years after the initial development of TMS, is often used as therapy. rTMS has controllable and repetitive frequency capabilities. It can be divided into high frequency (HF≥1 Hz; HF-rTMS) and low frequency (LF ≤ 1 Hz; LF-rTMS). These frequencies can either stimulate or inhibit the function of the cerebral cortex. rTMS can inhibit or stimulate unilateral brain function by regulating inter-hemispheric imbalanced inhibition. LF-rTMS was often used to inhibit the contralesional, unaffected hemisphere. HF-rTMS was often used to stimulate the ipsilesional, affected hemisphere (16). Both LF-rTMS and HF-rTMS were effective in the rehabilitation of motor dysfunction (17). The definite advantage of rTMS lies in its greater efficacy when applied during the acute and subacute phases, but not the chronic phase (18). Theta burst stimulation (TBS) is another mode of rTMS. It can efficiently reduce the stimulating time from 30 to 3 min. TBS uses two distinct stimulation methods: intermittent theta burst stimulation (iTBS) and continuous theta burst stimulation (cTBS).

#### 4.1.1 RTMS on upper limb function rehabilitation in stroke

Both LF-rTMS and HF-rTMS improved upper limb function in stroke patients in most of the studies, and it was interesting to note that LF-rTMS was more effective in contralesional, unaffected hemisphere compared with HF-rTMS in ipsilesional, affected hemisphere (19, 20). Most trials had two common points: combined therapy and small sample size. It made the effect of rTMS alone unclear (21, 22). Yuan et al. also highlighted that the protocol design of rTMS needed to be standardized to further clarify effectiveness. Although the independent validity of rTMS could not be confirmed, a meta-analysis revealed that combining physiotherapy with another rehabilitation therapy, such as a combination of occupational therapy and rTMS, yielded effectiveness (23). The combination of two physical therapies, viz., LF-rTMS and functional electrical stimulation could improve finger mobility and grip ability in chronic stroke patients (24); and the combination of LF-rTMS with neuromuscular electrical stimulation could improve upper limb function in the acute stage of stroke (25).

The use of LF-rTMS on the contralesional primary motor cortex was effective for hand motor recovery in the post-acute stage of stroke. This therapy reached level A evidence (18). Furthermore, patients with stroke who received rTMS in the acute stage were found to have improved upper limb function for more than 1 year at follow-up in a randomized study (26). Spasticity is another dysfunction of the upper limb following a stroke. Some researchers focused on it with rTMS. The effect of rTMS on the improvement of spasticity in stroke patients was controversial. The result of one meta-analysis showed no conclusive evidence of improvement in spasticity with rTMS (27). Most of the trials that showed improvement in spasticity were combined therapy (28, 29). For example, rTMS was combined with occupational therapy or repetitive facilitative exercise. RTMS was mostly used as a single therapy in trials that showed little therapeutic effect (30–32).

In brief, LF-rTMS is a good choice for hand motor recovery in the post-acute stage of stroke. Further research is needed to identify more optimal combination therapies through trials or meta-analyses. It remains unclear whether rTMS affects the improvement of spasticity in stroke patients.

#### 4.1.2 RTMS on lower limb function rehabilitation in stroke

Several meta-analyses have shown that rTMS could improve lower limb motor function in stroke patients (21, 33–35). LF-rTMS could improve spatial gait symmetry when applied for 10 sessions over a period of 2 weeks (36), and HF-rTMS could improve walking speed compared to LF-rTMS (37). There were several types of combined therapy, such as a combination of HF-rTMS or LF-rTMS with transcranial direct current stimulation (tDCS) (38, 39), a combination of LF-rTMS and motor relearning procedure (40), and a combination of HF-rTMS and treadmill training (41). These combined therapies were effective. However, it remained unclear which combined therapy was more effective. A meta-analysis also showed that tDCS was superior to rTMS in improving lower limb motor function except for the combined therapy (42).

RTMS might be effective in the recovery of lower limb function after stroke. It reached level B evidence in the guidelines (18). It was also noted that TBS was not recommended in terms of motor rehabilitation of stroke patients (18). Balance and ataxia are other important aspects of lower limb motor function. A preliminary study of LF-rTMS showed that 1 Hz rTMS over the cerebellum was safe (43). In subsequent studies, there was no clear and reliable evidence of whether rTMS could improve ataxia and balance after stroke in subsequent studies. However, we should be aware that a previous meta-analysis highlighted that tDCS was superior to rTMS in treating cerebellar ataxia (44).

In short, rTMS may impact lower limb recovery in stroke patients. Further research may reveal its impact on balance and ataxia, either in combination with other therapies or as a standalone treatment.

#### 4.1.3 RTMS on post-stroke aphasia

A 12-month, small-scale, and placebo-controlled trial showed that LF-rTMS had potential clinical application value in treating aphasia after stroke in 2011. The course of therapy was 10 days. The results mainly showed that LF-rTMS could improve naming, expression, and understanding abilities (45). Subsequently, a randomized, double-blind study also showed that LF-rTMS could improve the condition of patients with severe aphasia when applied to the right hemisphere's frontal language area, and the duration of the therapy was 20 days (46). The assessment was conducted 15 weeks after completing the therapy. It further suggested that rTMS could improve non-fluent aphasia after stroke in a 2017 meta-analysis (47). However, three randomized controlled trials indicated that LF-rTMS did not improve post-stroke aphasia in the short term (2–4 weeks) in 2019 (48–50). Several meta-analyses have shown that rTMS could improve post-stroke aphasia regardless of being combined with other treatments (50–54). These conclusions should be treated with caution due to the high heterogeneity and the lack of high-quality evidence. Therefore, controlled trials of rTMS in post-stroke aphasia with large samples and long observation periods were urgently needed (55, 56).

In brief, rTMS in post-stroke aphasia is promising, and a long duration of observation is needed. A large-scale, randomized, and controlled trial is needed to assess the effects of rTMS on post-stroke aphasia.

#### 4.1.4 RTMS on post-stroke dysphagia

The available results suggested that rTMS had a positive effect on post-stroke dysphagia (57–60), and HF-rTMS was usually chosen as therapy compared with LF-rTMS (61–64). The cerebellum is a common site to stimulate dysphagia. A randomized trial showed that rTMS combined with neuromuscular electrical stimulation could improve dysphagia in stroke patients and found that bilateral rTMS stimulation was better than unilateral stimulation (65). It differs from how rTMS treats motor dysfunction after stroke, which gives unilateral stimulation. However, a meta-analysis highlighted that there were still controversies about the best frequency and stimulated hemisphere (66). Bilateral cerebellar rTMS promoted the corticobulbar motor pathway to a greater extent than unilateral stimulation (67, 68). It was also found that unilateral and bilateral cerebellar stimulation of 10 Hz HF-rTMS could improve swallowing function by an observation of the treatment of dysphagia in brain-stem stroke patients. Bilateral stimulation could stimulate the excitability of the corresponding cortex more highly (61). Another sham-controlled double-blind trial showed that bilateral cerebellar stimulation with HF-rTMS improved dysphagia after stroke (69). Another single-blind randomized trial showed that 10 Hz rTMS at the bilateral motor cortex over the cortical areas projecting to the mylohyoid muscles was effective for dysphagia after stroke (70). It appears that rTMS holds more promise for dysphagia than for aphasia.

In recent years, TBS-related research in the field of stroke has gradually increased. A study found that iTBS could promote the excitability of the swallowing motor cortex and increase the connectivity of multiple brain regions in 2020 and might have therapeutic potential in treating dysphagia (71). A randomized controlled trial of 70 people showed that iTBS could improve dysphagia after stroke by stimulating the bilateral cerebellum, and it was safe in 2022. However, the effect mentioned in the article was better than rTMS, which remained to be verified (72). Another 47-person randomized controlled trial used HF-rTMS as the control group and found that there was no significant difference in clinical improvement and safety between iTBS and HF-rTMS. It suggested that iTBS might replace HF-rTMS in the field of dysphagia after stroke due to its efficiency in the same year. This trial also showed that iTBS was more effective than cTBS in improving dysphagia after stroke (73). However, the effectiveness still needed to be treated with caution due to a limited number of studies and heterogeneity (74).

In short, rTMS in the field of post-stroke dysphagia remains an area of significant interest. Dysphagia appears more promising for treatment with rTMS than aphasia, which may require a longer observation period for accurate assessment. It is suggested that either HF-rTMS or iTBS be selected to stimulate bilateral cerebellar regions. A large sample, randomized, and controlled trial should be conducted to determine the optimal combined therapy, which still needs to be further explored. Additionally, the long-term clinical effects and safety of rTMS for post-stroke dysphagia need to be further observed, as the optimal parameters and course of the treatment with rTMS remain uncertain.

#### 4.1.5 RTMS on post-stroke cognitive impairment patients

Vascular cognitive impairment (VCI) is the cognitive impairment caused by cerebrovascular diseases. Post-stroke cognitive impairment (PSCI) is one type of VCI. PSCI is also a hotspot, as shown in Figure 8. Therefore, this article also summarized the research of rTMS in the field of PSCI over the past 5 years.

A study found that 5 Hz rTMS and iTBS could both improve PSCI, and the effect of rTMS was more effective than iTBS as early as 2020 (75). A retrospective study in 2022 found that stimulation of the dorsal prefrontal cortex (DLPFC) on the ipsilesional, affected hemisphere using HF-rTMS (20 Hz) resulted in better cognitive improvement in patients compared with a blank control group (76). Subsequent trials showed that iTBS could improve cognitive dysfunction in stroke patients (77, 78). These trials all showed the same characteristic: small sample size. The results of several meta-analyses have confirmed the therapeutic effect of rTMS in PSCI; however, the recommended methods were controversial (79–82). Wang et al. believed that both LF-rTMS and HF-rTMS were effective for PSCI, and the combined use of LF-rTMS and HF-rTMS was more effective. ITBS was not superior to rTMS in effectiveness (83). Liu et al. believed that iTBS was the first choice and HF-rTMS was the second choice in improving PSCI and activities of daily living (ADL) (79).

On the contrary, Yang et al. concluded that HF-rTMS was preferred for improving cognitive impairment and ADL (84). It was suggested that both HF-rTMS and LF-rTMS effectively improved attention and memory impairments in PSCI patients but there was no significant difference between them (81, 85, 86). However, a meta-analysis indicated that rTMS combined with cognitive training did not improve memory impairment (87).

The efficacy of rTMS on PSCI exists based on the above results. However, the optimal stimulation mode and parameters for different cognitive impairments, such as memory and attention, have not yet been determined. It is possible that HF-rTMS and LF-rTMS are both effective in treating PSCI. However, the current results cannot confirm whether there is a difference in efficacy between iTBS and rTMS due to the heterogeneity.

In brief, rTMS for PSCI is a significant area of research with two main key issues: (1) the comparative efficacy of iTBS, HF-rTMS, and LF-rTMS as stimulation modes; (2) the definitive impact of rTMS on PSCI. Therefore, we advocate for larger-scale, randomized controlled trials to assess these methods and their safety in the future.

### 4.2 Bibliometric analysis of TMS in stroke

Scholars are expected not only to have a deep comprehension of their research field but also to maintain a broad understanding of evolving trends and interdisciplinary connections in today's era of big data. Bibliometrics provides comprehensive visualization and analysis, tracking the advancement of research across various fields. Furthermore, bibliometric analysis facilitates the exploration of emerging research areas and the identification of hotspots. It aids researchers in comprehensively and rapidly learning about the progress in TMS related to stroke. Moreover, it can indicate the direction for future research endeavors.

#### 4.2.1 The popularity of TMS research in the stroke field continues; the United States has the largest number of articles in this field

Research on TMS in the field of stroke has exhibited a significant rise in the number of publications between 1994 and 2023. Figure 2 shows the annual number of publications. It increased from 5 articles in 1994 to 314 articles in 2022. There were five articles in 1994 in the WoS core collection. The most highly cited publication from that year was Schnitzler and Benecke (88), with 111 citations, which was the highest among the 5 publications (88). The findings of this article also supported the notion that the silent period (SP) induced by TMS originated in the primary motor cortex. It provided theoretical support for the current treatment of stroke-related disability using TMS.

Although there were declines in the number of articles in adjacent years (2008–2009, 2017–2018, and 2022–2023) in Figure 2, the overall trend still indicated an increasing number of publications. Figure 3 illustrates that the application of TMS in stroke initially originated in Europe, particularly in Germany and the United Kingdom before gradually expanding to the United States and other countries in the Americas. The number of relevant articles published by China has significantly increased, indicating a literature burst since 2020. According to Table 1, the United States has published the most (953 articles), closely followed by China (546 articles). This indicated that although China started later than others, it has been actively engaging in more pertinent research in recent years.

#### 4.2.2 Harvard University had the most articles on TMS in the field of stroke

Four institutions, including Harvard University, NINDS, Emory University, and Northwestern University, are located in the United States. Three institutions are located in Europe (University College London, the University of Manchester, and University Tubingen). Two institutions are located in Asia (Yeungnam University and JiKei University). One institution is located in Oceania (University of Auckland) and is among the top 10 institutions that have made contributions. Four institutions in the United States specialized in using TMS in stroke research, as previously shown in Table 2. The institution with the largest proportion of articles was Harvard University (138 articles, 12,813 citations). It accounted for 19.1% of the total national publications and 24.3% of the total national citations. The remaining 3 institutions were NINDS with 65 articles. Emory University has 61 articles, and Northwestern University has 58 articles. Table 2 and Figure 6 highlight two institutions, namely, Harvard University and University College London, with centrality >0.1. It indicated that these two institutions played a pivotal role and were potential partners for other institutions in this field. China (402 articles) ranked second in the country of publication. However, no Chinese institution published more articles than the University of Manchester, which had 58 articles ranked 10th. This might be due to the involvement of many research institutions in China, each publishing a relatively small number of articles.

The search result showed that Harvard University had published 81 articles over the past 10 years (2014–2023). TMS was used as a therapy for motor recovery (37, 89, 90). Furthermore, these results suggested that rTMS might effectively improve motor function. However, the results of Harvey et al. showed no significant improvement in upper limb function after stroke. This might be because the intervention was not conducted during the acute stage of stroke when rTMS is more effective. In addition, two studies demonstrated the safety of rTMS application on stroke patients and its potential to improve motor function (91, 92).

In brief, Harvard University's research in the field of TMS has been extensive. The sample is larger and of higher quality, making it suitable for new researchers to learn from. However, the number of related articles has been decreasing in recent years.

University College London published 68 articles between 2014 and 2023, during which TMS was primarily used to evaluate stroke neuropathology (93–97). In addition, it also provided theoretical support for the treatment of post-stroke dysphagia and aphasia with rTMS. Sasegbon et al. hypothesized that stimulating the bilateral cerebellar pathway with rTMS was more effective than a unilateral approach for post-stroke dysphagia. The result showed that an increase in cortical excitability associated with pharyngeal movements when 10 Hz rTMS stimulated the cerebellum bilaterally or unilaterally (67, 98, 99). This was a reason for the increase of rTMS research on post-stroke dysphagia in recent years. Therefore, TMS has the potential to be used as a therapy for dysphagia. The reference with the most citations from University College London was “Evidence-based guidelines on the therapeutic use of repetitive transcranial magnetic stimulation (rTMS)” in 2014. This article summarized the various fields of rTMS application and provided evidence for the effect on dysfunction or disease. There was another updated version in 2020, and the details were discussed in Section 4.1.

NINDS and the University of Tubingen experienced the earliest literature burst. They concentrated around 2010 as shown in Figure 5 followed by Harvard University, University College London, and University Manchester. This also indicated that the United States made rapid progress in this field. In recent years, literature burst in this field primarily focused on China. Sun Yat-sen University was one of the representatives.

Sun Yat-sen University has published a total of 50 articles. Most of them have focused on the field of post-stroke dysphagia and aphasia over the past 10 years. The main research findings were as follows. A single-blind randomized controlled trial showed that 10 Hz HF-rTMS delivered under the cerebellum could improve infratentorial stroke dysphagia (68). Another randomized, double-blind, controlled trial showed iTBS could also improve post-stroke dysphagia (72). A study showed that 5 days might be the shortest treatment duration for post-stroke dysphagia (66). In addition to clinical trials, there were studies on the mechanism of rTMS in stroke therapy. As early as 2017, some studies highlighted that HF-rTMS could be used to improve functional recovery in patients with ischemic stroke by enhancing neurogenesis and activating brain-derived neurotrophic factor (BDNF) and tropomyosin-related kinase B (TrkB) signaling pathways (100). RTMS could modulate microglia with anti-inflammatory polarization variation. It could also promote neurogenesis and the proliferation of neural stem cells in subsequent studies for ischemic stroke (101, 102). For the mechanistic study of dysphagia, two studies showed that HF-rTMS could modulate the composition of gut microbiota and improve aspiration-induced pneumonia caused by dysphagia (103, 104). In short, if an individual is interested in rTMS in the therapy of post-stroke dysphagia, they may follow the work of Sun Yat-sen University.

#### 4.2.3 The cooperation between the authors from different countries needs to be further strengthened

Among the top three productive authors listed in Table 3, only Abo, Masahiro is from Japan, while, Fregni, Felipe, and Pascual-Leone, Alvaro, are from the United States. Abo, Masahiro's research has primarily focused on clinical research in this field over the past 3 years. “NEURO” was the recommended therapy in these trials, where LF-rTMS was combined with one-on-one intensive occupational therapy. The advantage of this combination therapy was its ease of implementation. The results of several retrospective studies showed that “NEURO” therapy was helpful for upper limb functional recovery after stroke. The effect of recovery was related to the severity of the stroke (105, 106). The mechanism of LF-rTMS combined with occupational therapy enhanced the functional roles of networks in motor-related areas of the ipsilesional cerebral hemisphere in the latest study (107).

Additionally, other studies have shown that the application of HF-rTMS in the acute phase of stroke is safe (108, 109). A literature study focused on the use of HF-rTMS in combination with intensive speech-language-hearing therapy for aphasia. It was found that HF-rTMS combined with speech therapy had a positive effect on both fluent and non-fluent aphasia. However, the results require further validation through studies with large sample sizes (110). It should be noted that Abo, Masahiro's clinical studies had small sample sizes (< 100 people on average). His research team also developed a protocol in 2022 (111) indicating the need for a large, multicenter, controlled study in this field. However, the related article has not yet been published.

In summary, Abo Masahiro's team has conducted in-depth research on rTMS in combination with occupational therapy. This combined therapy is very suitable for rehabilitative therapists or hospital clinical practitioners to carry out and observe the results. These results are also very suitable for new researchers to learn about, but the drawback is that the independent effects of rTMS cannot be observed.

Fregni, Felipe is affiliated with Harvard University. Fregni, Felipe conducted a TMS study with fewer publications in recent years compared to Abo Masahiro. Fregni, Felipe demonstrated using TMS as a single therapy often and combined therapy less frequently to observe the results in the experimental design. Furthermore, the sample size of Fregni, Felipe's team was larger in this field than Abo Masahiro. Interestingly, Fregni designed a study to investigate biomarkers associated with functional disability in a 2021 protocol article. The study utilized evaluation tools such as TMS electroencephalograms, functional near-infrared spectroscopy, and magnetic resonance imaging. The content is worth paying attention to in the future (112).

Compared with Fregni, Felipe, Pascual-Leone, Alvaro has published fewer relevant articles in recent years. There were mainly three related studies, all published in 2016. One study evaluated the improvement of assisted robots in stroke patients with rTMS (113). The other two studies explored the effects of LF-rTMS and HF-rTMS on motor function in stroke patients. The results also indicated that LF-rTMS could enhance cortical excitability and the response of the affected hand in the ipsilateral hemisphere (92). HF-rTMS intervention should be individualized based on functional corticospinal tract status and brain-derived neurotrophic factor genotype to improve the upper extremity motor of patients with stroke (114).

Although Abo, Masahiro published the most articles, with 52 articles and 1,002 citations, the citations were significantly lower than those of Fregni, Felipe (4,776 citations) and Pascual-Leone, Alvaro (5,151 citations). The centralities of individual authors are < 0.1. This indicates that authors should enhance collaboration between countries.

It could be observed from Table 3 that two authors, Rossini, PM, and Khedr, EM, had centrality values >0.1 in terms of co-citation. The co-citations of Khedr, EM's literature did not reach those of Rossini, PM. Khedr, EM mostly focused on LF-rTMS or HF-rTMS in stroke patients' motor function recovery (115–118) and dysphagia (119–121). Rossini, PM (122, 123) had few studies published in this area. His relevant articles served as guidelines for the clinical application of rTMS, which might also explain its high centrality (135).

#### 4.2.4 The United States accounts for the largest proportion of the top 10 publications in this field

Three publishing houses are based in the USA, and two are located in the UK. Three publishing houses are located in Switzerland. One publishing house is in the Netherlands and the other in Germany. There are three publishing houses located in quartile one of the JCR divisions: *Neurorehabilitation and Neural Repair, Stroke*, and *Brain Stimulation*. There are three in quartile two, including *Clinical Neurophysiology, Frontiers in Neurology*, and *Frontiers in Neuroscience*. There are two in quartile three: *Frontiers in Human Neuroscience, Restorative Neurology, and Neuroscience*. The remaining publishing house is located in quartile four: *Experimental Brain Research*. A total of 882 articles were published, with American publishing houses contributing the largest share, accounting for 31.1% of the top 10 publishing houses' outputs. The journal *Brain* had the highest impact factor among the top 10 journals. It had an impact factor score of 14.5 points. The remaining nine journals did not exceed 10 points; their H-index reached 365. However, only 50 articles were included. The research content in the Journal *Brain* was mostly neurophysiological research after stroke. TMS was used as an assessment. Some studies highlighted that there was a disinhibition of the motor cortex in the unaffected hemisphere and an exaggeration of inhibition in the affected hemisphere after a stroke (124, 125). Through 2 years of observation, Classen et al. found that when TMS was applied to stimulate the contralateral hand muscles of the affected hemisphere, it resulted in an SP extension and a normal evoked potential. Hyperactivity of cortical inhibitory interneurons might be the reason for the motor dysfunction observed in stroke patients. Netz et al. found that the TMS in affected or unaffected hemispheres could extend the SP, and motor output was reorganized in the non-affected hemisphere. However, Netz et al. did not consider this reorganization of motor output to be clinically improved (126). Another study also noted that cortico-bulbar tract fibers were involved in dysarthria after stroke (127). This is also a referential location to stimulate TMS for dysfunction after stroke, such as dysphagia and aphasia. Hummel et al. (128) did not propose TMS as a rehabilitation treatment for stroke until 2005 in the Journal *Brain*. The results also mentioned that TMS might be used as an adjunct to neurorehabilitation. However, this did not mean that Hummel, F. was the first to introduce TMS into the field of stroke rehabilitation. There were five articles included in 2012. However, the number of literature included decreased, with an average annual inclusion of 1.45 articles (2013–2023).

There were 65 related articles in the journal *Stroke*, whose impact factor ranked second, and the content was mostly related to the prognosis of TMS in the therapy of stroke. The study by Traversa et al. highlighted that there was plasticity after central nervous system injury in adults and that the plasticity could persist for 2–4 months (129). A study suggested that inter-hemispheric asymmetry of the motor cortex was associated with stroke recovery, which was a mechanism for rTMS in stroke (130). It was found that TMS could induce motor evoke potential (MEP) in stroke patients, and MEP had been proposed to have a prognostic effect (131, 132). MEP has also been used in subsequent TMS-related studies. In short, these findings provide a theoretical basis for studying TMS in stroke.

The earliest article applying transcranial magnetic stimulation (TMS) technology to stroke rehabilitation, authored by Arac et al. in 1994, primarily explored the prognostic value of TMS in stroke treatment. The outcome of this study was negative, which can be attributed to the fact that repetitive TMS (rTMS), as it is used today, had not yet been fully developed for stroke rehabilitation at that time. To validate our findings, we conducted an extensive search within the Web of Science (WoS) database, including all relevant databases and collections, dating back to 1988. The first research result related to TMS therapy for stroke remains the study by Arac et al. (133). Notably, six articles in this field were published in 2006 in the journal *Stroke*, and the average annual number of articles published in the past decade (2013–2023) was 2.7.

#### 4.2.5 Three articles have played significant roles in the field

The most citations were 1,578 times, and the least were 794 times. It was noteworthy that the top three articles, all published after 2010, were guidelines: Langhorne et al. (7), Winstein et al. (134), and Lefaucheur et al. (123). The most cited article was by Langhorne et al. (7). In the article by Langhorne et al. (7), TMS was applied in stroke rehabilitation, and the authors acknowledged that it was still uncertain whether these interventions enhanced functional outcomes. There might be advancements in combination therapy in these fields. It was confirmed that combination therapy was effective in Winstein et al. (134). The article noted that brain stimulation technology, including TMS, could have therapeutic effects when combined with behavioral or language therapy. This could explain why aphasia has become a therapeutic hotspot for rTMS treatment in recent years. It should be noted that Winstein et al. (134) had 1,349 citations, while the publication date was as recent as 2016.

#### 4.2.6 The applications of rTMS in dysphagia and cognitive impairment of stroke may be future hotspots

As shown in Table 5, the top three keywords with the highest frequency of occurrence based on this search query string were as follows: “stroke” (1,119 times), “transcranial magnetic stimulation” (1,030 times), and “rehabilitation” (387 times). The frequency of “rehabilitation” was significantly lower than that of the first two keywords. It could be observed that the peak period for the keywords “stroke” and “TMS” was relatively close, occurring around 2014, as shown in Figure 8. Subsequently, the research focus shifted to rTMS (around 2016), which was mostly used for motor recovery, aphasia, and dysphagia in stroke patients. In recent years, the focus of research has included rTMS therapy for aphasia and dysphagia. It was found that 335 articles were retrieved using “aphasia” as the keyword in the search results. The average number of relevant articles published per year was 23.5 over the past 10 years (2013–2023). Most of the articles were published in 2022 (28 articles). When dysphagia was retrieved as a keyword, it could be observed that the number of relevant articles was significantly lower than that of aphasia (166 articles). However, surprisingly, there was an explosive growth in the number of relevant articles in 2022 (38 articles). It was the year with the highest number of articles published on dysphagia following a stroke. The number of articles on dysphagia surpassed that on aphasia (2021–2023). This indicated that dysphagia has been emerging as a research hotspot in the field of stroke rehabilitation over the past 3 years (2021–2023). Thus, treatment of dysphagia may be a hotspot by rTMS in the future.

Research in the field of cognitive impairment related to stroke has been increasing in the last 3 years, as shown in Figure 8. This trend indicated that treating PSCI by rTMS may be another hotspot. The related literature volume was up to 103 articles. The type of article included protocols, clinical observations, and meta-analysis. The details are discussed in Section 4.1.5.

## 5 Conclusion

In summary, TMS research in the field of stroke continues to be active and promising, with the United States leading in the number of published articles at 953. Harvard University and University College London have demonstrated significant betweenness centrality, highlighting their pivotal roles and potential as key collaboration partners. Strengthening author cooperation across countries is advisable. TMS applications for post-stroke cognitive impairment, aphasia, and dysphagia are emerging as research hotspots with promising prospects. Combining rTMS with occupational therapy may offer potential benefits for upper limb recovery after a stroke. Identifying more effective combined therapies with rTMS remains a priority. Future research should focus on large-scale, randomized, and controlled trials to address these post-stroke dysfunctions.

## 6 Limitations

There are some limitations to our study. First, it takes a certain amount of time for an article to achieve a certain number of citations. High-quality literature needs to take time to reach the expected citations. Second, VOSviewer does not list the affiliated organization and the WoS division organization is more detailed than VOSviewer. As a result, the overall statistical results of VOSviewer are lower than the actual data and the synonym replacement function cannot be completely covered. Therefore, the corresponding statistical data shall be subject to the data displayed in WoS. Third, we limited our analysis to English language articles, which may have led to the exclusion of non-English high-quality literature. Finally, in the author analysis, we did not distinguish between the first author and other authors, which could have affected the interpretation of the author's impact.

## Data Availability

The raw data supporting the conclusions of this article will be made available by the authors, without undue reservation.
