# Supplementary material for: Global research trends in transcranial magnetic stimulation for stroke (1994–2023): promising, yet requiring further practice
Source: Front Neurol. 2024 Aug 29;15:1424545. doi: 10.3389/fneur.2024.1424545 (PMC11390666; doi:10.3389/fneur.2024.1424545)
Supplement: Supplementary file 1 [file Table_1.docx]

#### Supplementary Table1.Top 10 cited article related to the research of TMS on Stroke

| Rank | Title | Journal | Author | Citiation | Year |
| --- | --- | --- | --- | --- | --- |
| 1 | Stroke Care 2 Stroke rehabilitation | *Lancet* | Langhorne,Peter et al. | 1578 | 2011 |
| 2 | Guidelines for Adult Stroke Rehabilitation and Recovery A Guideline for Healthcare Professionals From the American Heart Association/American Stroke Association | *Stroke* | Winstein,Carolee J et al. | 1555 | 2016 |
| 3 | Evidence-based guidelines on the therapeutic use of repetitive transcranial magnetic stimulation (rTMS) | *Clinical Neurophysiology* | Lefaucheur,Jean-Pascal et al. | 1289 | 2014 |
| 4 | Transcranial DC stimulation (OCS): A tool for double-blind sham-controlled clinical studies in brain stimulation | *Clinical Neurophysiology* | Gandiga,PC et al. | 1253 | 2006 |
| 5 | The plastic human brain cortex | *Annual Review Of Neuroscience* | Pascual-Leone,A et al. | 1098 | 2005 |
| 6 | Influence of interhemispheric interactions on motor function in chronic stroke | *Annals Of Neurology* | Murase,N et al. | 1054 | 2004 |
| 7 | Treatment-induced cortical reorganization after stroke in humans | *Stroke* | Liepert,J et al. | 953 | 2000 |
| 8 | Evidence-based guidelines on the therapeutic use of repetitive transcranial magnetic stimulation (rTMS): An update (2014-2018) | *Clinical Neurophysiology* | Lefaucheur,Jean-Pascal et al. | 842 | 2020 |
| 9 | Effects of non-invasive cortical stimulation on skilled motor function in chronic stroke | *Brain* | Hummel,F et al. | 835 | 2005 |
| 10 | A practical guide to diagnostic transcranial magnetic stimulation: Report of an IFCN committee | *Clinical Neurophysiology* | Groppa,S et al. | 794 | 2012 |

**Supplementary Table 2.** **Top 10 co-cited article related to the research of TMS on Stroke**

| Rank | Title | Journal | Author | Year | Citiation | Total link-strength |
| --- | --- | --- | --- | --- | --- | --- |
| 1 | Influence of Interhemispheric Interactions on Motor Function inChronic Stroke | *Annals of Neurology* | Murase ,N et al. | 2004 | 499 | 15764 |
| 2 | Safety,ethical considerations,and application guidelines for the use of transcranial magnetic stimulation in clinical practice and research | *Clinical Neurophysiology* | Rossi S et al. | 2009 | 448 | 8959 |
| 3 | Non-Invasive Electrical And Magnetic Stimulation Of The Brain,Spinal Cord And Roots: Basic Principles And Procedures For Routine Clinical Application.Report Of An Ifcn Committee | *Electroencephalography and Clinical Neurophysiology* | Rossini PM et al. | 1994 | 371 | 6367 |
| 4 | The Assessment and Analysis of Handedness: The Edinburgh Inventory | *Neuropsychologia* | Oldfield RC et al. | 1971 | 324 | 5008 |
| 5 | Theta Burst Stimulation of the Human Motor Cortex | *Neuron* | Huang YZ et al. | 2005 | 313 | 7812 |
| 6 | Repetitive Transcranial Magnetic Stimulation of Contralesional Primary Motor Cortex Improves Hand Function After Stroke | *Stroke* | Takeuchi N et al. | 2005 | 298 | 9710 |
| 7 | Effects of non-invasive cortical stimulation on skilled motor function in chronic stroke | *Brain* | Hummel F et al. | 2005 | 291 | 9279 |
| 8 | Excitability changes induced in the human motor cortex by weak transcranial direct current stimulation | *Journal of Physiology-London* | Nitsche MA et al. | 2000 | 291 | 8616 |
| 9 | A sham stimulation controlled trial of rTMS of the unaffected hemisphere in stroke patients | *Neurology* | Mansur,CG et al. | 2005 | 283 | 9044 |
| 10 | The post-stroke hemiplegic patient.1.a method for evaluation of physical performance. | *Scandinavian Journal Of Rehabilitation Medicine* | Fuglmeyer AR et al. | 1975 | 276 | 5157 |
